# Simultaneous Determination of Tetrodotoxin in the Fresh and Heat-Processed Aquatic Products by High-Performance Liquid Chromatography–Tandem Mass Spectrometry

**DOI:** 10.3390/foods11070925

**Published:** 2022-03-23

**Authors:** Hongli Ye, Yinfeng Xi, Liangliang Tian, Dongmei Huang, Xuanyun Huang, Xiaosheng Shen, Youqiong Cai, Yuan Wangs

**Affiliations:** 1 Laboratory of Aquatic Product Quality, Safety and Processing, East China Sea Fisheries Research Institute, Chinese Academy of Fishery Sciences, Shanghai 200090, China; yehongli12@163.com (H.Y.); xiyinfeng@126.com (Y.X.); tian-19860120@163.com (L.T.); hdm2001@126.com (D.H.); hxyseven@163.com (X.H.); foodsmc98@126.com (X.S.); caiyouqiong@163.com (Y.C.); 2 Key Laboratory of Control of Safety and Quality for Aquatic Product, Ministry of Agriculture and Rural Affairs, Beijing 100141, China

**Keywords:** simultaneous determination, tetrodotoxin, high-performance liquid chromatography–tandem mass spectrometry, *Takifugu*, heat-processed *Gadus*

## Abstract

Tetrodotoxin (TTX) was simultaneously detected in the fresh and heat-processed aquatic products by high-performance liquid chromatography–tandem mass spectrometry method. The detection conditions were investigated, including the chromatography column and mobile phase. Based on the optimized parameters, a sensitive determination method of TTX was established. The proposed method featured the merits of a good linear relationship between signal and TTX concentration (R^2^ = 0.9998), a wide detection matrix-based range of 0.2–100 ng/g, and a low detection limit of 0.2 ng/g, etc. The spiked assays evidenced its accuracy and reliability with recoveries of 90.5–107.2%. Finally, the developed method was simultaneously successfully applied in the determination of TTX in various fresh and heat-processed aquatic products.

## 1. Introduction

As a neurotoxic drug, tetrodotoxin (TTX) has been widely applied in cancer treatments, analgesics, antibacterial and anti-inflammatory applications, enhancement the anesthetic effects of narcotic drugs for its advantages of low-molecular weight, non-protein, and stability in high temperatures [1,2,3,4]. However, TTX has been confirmed a lethal toxin for aquatic products or mice owing to the abilities of blocking the voltage-gated sodium channels in nerve and muscle tissue, inhibiting the production of the action potential, and paralyzing the functions of nerve and muscle [5,6,7]. Moreover, TTX has been proved to enter into the human body through the food chain, suffer many food poisoning incidents, and endanger human life [8,9,10,11]. Hence, the reported TTX-containing aquatic products, such as pufferfish [12,13,14,15], the blue-lined octopus [16], marine bivalves [17,18], and crab [19], etc. have caught more attention in consumption and diet. The deliciously heat-processed aquatic products are the most common fish products in China, generally obtained from boneless fish fillets by a series of processing steps [20]. The level of toxins in heat-processed fish is of significant importance, however, to our knowledge, TTX existing in processed aquatic products have not been reported. Therefore, it is necessary to propose a sensitive and accurate method to detect TTX to ensure the quality and safety of the processed aquatic products to avoid the harm caused by the TTX.

A variety of TTX detection approaches have been extensively developed by mouse bioassay [21,22], fluorescence sensing method [23,24,25,26], electrochemical analysis [27], immunosorbent assay [28,29,30,31], gas chromatography/mass spectrometry (GC/MS) [32,33], high-performance liquid chromatography/mass spectrometry (HPLC-MS/MS) [34,35], and so on. The mouse bioassay has the disadvantages of time consumption, exertion, and poor repeatability. At the same time, the fluorescence sensing method and immunosorbent assay possessed the defects of low efficiency and impracticality, which influenced the determination of TTX in complex samples based on these approaches [36]. The polar and nonvolatile intrinsic properties of TTX restrict its detection by GC/MS method. HPLC-MS/MS technology overcoming these disadvantages has been drawn substantial attention due to its merits of facilely rapid response, good sensitivity, high accuracy, strong separation ability, and extensive application in complex samples [37].

Herein, we developed a highly sensitive determination method of TTX based on HPLC-MS/MS technology. In order to get the best detection effect, the conditions including the chromatography columns and the mobile phase were investigated. The presented method was validated by the linear detection range (LR), the limit of detection (LOD), and spiked assays, etc. Finally, the proposed method was simultaneously applied in the determination of TTX in various fresh and heat-processed aquatic products.

## 2. Materials and Methods

### 2.1. Samples

The 16 fresh pufferfish samples (e.g., T. obscurus, T. flavidus, and T. rubripes) were provided from the provinces of Fujian, Guangdong, Shanghai, Shandong, Jiangsu, Hebei, which were dissected into the skin, muscle, liver, or gonad tissues. Each tissue was washed with water to remove blood stains, cut into pieces, and homogenized, before being kept at −20 °C for further treatment. The 26 heat-processed aquatic products were provided by the markets in Shanghai, which were also homogenized and stored at −4 °C, such as heat-processed Trichogaster leeri, heat-processed Gadus, heat-processed Anguillidae, heat-processed Redeye mullet, heat-processed Thamnaconus septentrionalis, heat-processed Scomberomorus niphonius, heat-processed Lophiiformes and heat-processed Rajiformes.

### 2.2. Materials and Instruments

Methanol (MeOH, HPLC grade) and acetonitrile (ACN, HPLC grade) were purchased from Avantor^TM^ Performance Materials Corporation (J. T. Baker, Phillipsburg, NJ, USA). Formic acid (FA, HPLC grade), acetic acid (ACA, HPLC grade), and ammonium acetate (AMA, HPLC grade) were provided from Shanghai Aladdin Biochemical Technology Co., Ltd. (Shanghai, China). 10 mM of phosphate-buffered saline buffer (PBS) was from Beijing Soleibao Technology Co., Ltd. (Beijing, China). Ultrapure water (18.2 MΩ·cm^−1^) produced from a Milli-Q EQ 7000 (Millipore, Billerica, MA, USA) was used throughout the experiments. The certified TTX standard (C_11_H_17_N_3_O_8_, ≥98%) was provided from the Affixscientific Corporation (Fremont, CA, USA). Tetrodotoxin immunoaffinity column solid-phase extraction (SPE) cartridges (3 mL/1000 ng) were supplied from Jiangsu Meizheng Biological Technology Co., Ltd. (Wuxi, China). A tripe-quadrupole mass spectrometer UltiMate3000/TSQ Quantum ACCESS equipped with a high-performance liquid chromatography system purchased from Agilent Technologies Co., Ltd. (Thermo Scientific, San Jose, CA, USA) was used for the determination of TTX.

### 2.3. Sample Pretreatment

Homogenized samples of 5.00 (±0.02) g were accurately weighed in the 30 mL of centrifuge tubes, then 10 mL of 1% acetic acid-methanol solution was added into the tubes. After being shaken vigorously, the stock solutions were treated with ultrasound at 40 °C for 15 min and subsequently centrifuged at 10,000 r/min for 10 min. 7 mL of the obtained supernatants and 28 mL of PBS were added into a 50-mL centrifuge tube. Mixed thoroughly later, the pH values of the obtained solutions were adjusted to be 7.0 by 2 M of sodium hydroxide solution. The immune affinity columns were utilized to purify the sample solutions. 10 mL of the precursor was eluted with 4 mL of a 2% acetic acid–methanol solution. The eluent was dried at 40 °C in nitrogen gas, and 1.0 mL of 0.05% formic acid–acetonitrile solution was adopted to dissolve the residue, and subsequently measured by HPLC-MS/MS. Each real sample was performed in parallel.

### 2.4. HPLC-MS/MS Conditions

An Acquity UPLC BEH Amide column (150 mm × 2.1 mm, 3.5 μm, Waters, Wexford, Ireland) was used to separate TTX. The values of flow rate, injection volume, and column temperature were set to be 0.3 mL/min, 10 μL and 40 °C, respectively. 0.1% formic acid aqueous solution and pure acetonitrile solution were used as mobile phase A and B, respectively. The gradient elution conditions were performed step by step, as listed in Table 1.

TTX was acquired in a selected reaction monitoring (SRM) positive ionization mode in electrospray source ion (ESI) mode at a spray voltage of 4800 V. The ion transport tube temperature was set to 350 °C. Ar was utilized as a collision gas with the pressure of 1.5 m Torr. The sheath gas and auxiliary gas were set to the appropriate parameters. The Q1 mass fragment of TTX was explored to 320.2 *m*/*z*, and the Q3 mass fragments to 302.2 *m*/*z*, 162.2 *m*/*z*, 284.2 *m*/*z*, whose collision energies were employed to 26, 36, and 24 eV, respectively.

### 2.5. Method Validation

The proposed method was validated by LR, calibration curves (CV), correlation coefficient (R^2^), LOD, the limit of quantitation (LOQ), repeatability, matrix effect (*ME*), and accuracy, etc. LOD and LOQ were determined from the matrix-based calibration curve at a signal-to-noise ratio of 3 and 10 according to the reported literature [38,39]. Repeatability was calculated for twenty replicates of 20 ng/g TTX in the *T. obscurus* and heat-processed *Gadus* samples. *ME* was tested by the solvent-based calibration curves and the matrix-based calibration curves on the basis of Equation (1) [40].

(1) $$ME\;\left(\%\right)=\left(1-\frac{S_{solvent}}{S_{matrix}}\right)\times 100\%$$

where *S_solvent_* and *S_matrix_* were the slopes of the solvent-based calibration curve and matrix-based calibration curve, respectively. The calibration curves were plotted by the chromatographic peak areas and the corresponding concentrations of TTX standard solutions. Meanwhile, the matrix-based calibration curve was acquired from the spiking standard solutions after the pretreatment. The value of *ME* implied an ionization enhancement (less than 0), an ionization suppression (more than 0), or no influence (equal to 0).

Spiked assays were utilized to evaluate the accuracy, repeatability and feasibility of the developed method. The detailed experiments were conducted as follows. Briefly, 0.5 ng/g, 2.5 ng/g, and 10 ng/g of TTX were added into the TTX-free heat-processed *Gadus* and the different tissues of TTX-free fresh *T. obscurus*, for instance, skin, muscle and liver. Each sample was measured in six parallel tests to ensure the reliability of the results.

### 2.6. Statistical Analysis

All the figures in the work were drawn by Origin 2021 software. Microsoft Excel 2019 was utilized for statistical analysis, including the calculation of the spiked recovery, the relative standard deviations, and etc.

## 3. Results and Discussion

### 3.1. HPLC-MS/MS Conditions

The parent of TTX was a measure to 320.2 *m*/*z* in our work, which was broken up into several product ions. Figure S1 shows the total ion chromatogram (TIC) and the selective product ions chromatograms of TTX with a concentration of 25 ng/mL. The results indicated that the appropriate product ions of TTX were 320.2 > 302.2 *m*/*z* ((corresponding to a loss of water, C_11_H_15_N_3_O_7_), 320.2 > 162.2 *m*/*z* (C_9_H_8_N_3_O), and 320.2 > 284.2 *m*/*z* (C_11_H_14_N_3_O_6_), which were consistent with those reported in the previous studies [41,42]. Moreover, among the three identified product ions, 320.2 > 302.2 *m*/*z* displayed the considerable superiority, which was considered as the quantitative ion during the determination of TTX for its high intensity.

**Figure 1:**
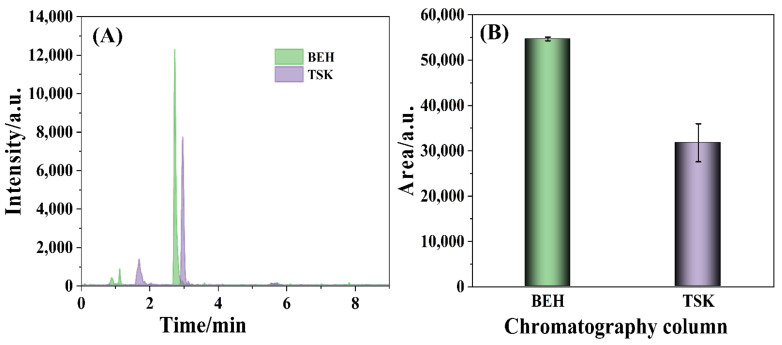
The chromatographic peaks (**A**) intensity and (**B**) area of the 320.2 > 302.2 *m*/*z* ion fragment detected by the BEH amide column and TSK amide column with a TTX concentration of 25 ng/mL.

TTX is a water-soluble and strong polarity biotoxin, which results in the poor ability of retention on the reversed-phase column (e.g., C18). Amino chromatographic column was compatible to detect TTX for the carbamoyl-bonded silica gel filler, suitable polarity, good retention, high response, etc. [43]. TSK gel Amide-80 column (150 mm × 2.0 mm, 5 μm, TOSOH, Honshu, Japan) was specified to measure TTX in the national standard (GB 5009.206-2016). Herein, the comparison of the TTX determination by BEH amide column and by TSK amide column was carried out, which was depicted in Figure 1. The retention time of TTX was 2.75 min detected by BEH amide column, which was almost the same as 2.97 min by the specified TSK amide column (Figure 1A). However, it was noteworthy that the intensity of the 320.2 > 302.2 *m*/*z* ion fragment measured by BEH amide column was significantly higher than that by TSK amide column. Meanwhile, the chromatographic peak area of the 320.2 > 302.2 *m*/*z* ion fragment detected by BEH amide column was distinctly larger than that by TSK amide column (Figure 1B). The results manifested the good performance of BEH amide column for the detection of TTX.

**Figure 2:**
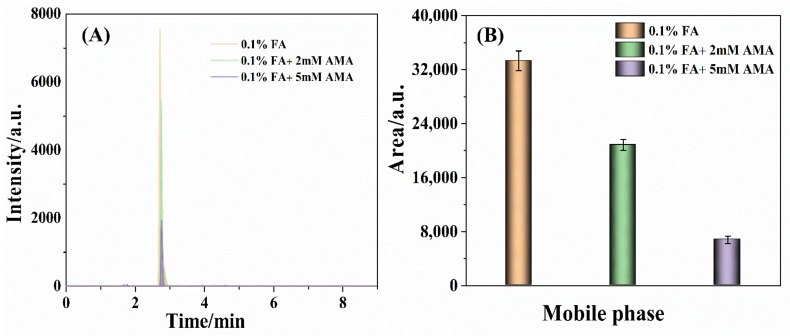
The chromatographic peaks (**A**) intensity and (**B**) area of the 320.2 > 302.2 *m*/*z* ion fragment detected by the BEH amide column taking 0.1% FA aqueous, 0.1% FA+ 2 mM AMA aqueous, 0.1% FA + 5 mM AMA aqueous as the mobile phase, respectively, with TTX the concentration of 25 ng/mL.

AMA could improve the ionization efficiency of the target compound and be used as the additive in the mobile phase. The national standard for TTX detection (GB 5009.206-2016) used 0.1% FA aqueous solution containing 5 mM AMA as mobile phase. Rodríguez, I. et al. utilized 0.1% FA aqueous solution containing 10 mM AMA as a mobile phase to determine the toxins in shellfish tissue [34]. Ochi, N. employed the 5 mM ammonium formate aqueous solution as the mobile phase to detect the toxins in bivalve samples, which was adjusted the pH value to 3.5 with FA reagent [44]. To discuss the effect of AMA on the determination of TTX, 0.1% FA aqueous, 0.1% FA aqueous containing 2 mM AMA (0.1% FA + 2 mM AMA), and 0.1% FA aqueous containing 5 mM AMA (0.1% FA + 5 mM AMA) were applied as the mobile phase in this work, respectively. As shown in Figure 2A, the retention time of TTX was scarcely changed as the AMA concentration gradually increased from 0 mM to 5 mM. Remarkably, the intensity of the 320.2 > 302.2 *m*/*z* ion fragment was sharply decreased with the concentration of AMA increasing from 0 to 5 mM. Furthermore, the peak area of the 320.2 > 302.2 *m*/*z* ion fragment dramatically descended with the AMA concentration enlarging from 0 to 5 mM (Figure 2B). The results indicated that the 0.1% FA aqueous without AMA was an appropriate mobile phase to measure TTX.

### 3.2. Method Validation

Under the optimum conditions, the determination method of TTX was established. Table 2 lists the LR, CV, LOD, LOQ and *ME* of the proposed method. It was observed that the values of *ME* in *T. obscurus* and heat-processed *Gadus* were 18.3% and −0.671%, which indicated the negligible ionic suppression in *T. obscurus* sample and ionic enhancement in heat-processed *Gadus* sample during the TTX detection (*ME* less than ±20% [45]). Meanwhile, the relationship between the area and concentration of TTX showed a satisfactory linear (R^2^ > 0.99) with the concentration of TTX varying from 0.2 to 100 ng/g. LOD and LOQ were measured to be 0.2 ng/g (S/N > 3), and 0.5 ng/g (S/N > 10), respectively, which were lower than those reported in the National Standard of China (GB 5009.206-2016: 1.0 and 3.0 ng/g). The results manifested the high performance of the developed method for TTX detection. Moreover, Table 3 further evidences the excellent high sensitivity of the presented method for the TTX determination in aquatic products in comparison with the previously reported literature.

As presented in Table 4, the recoveries of 0.5 ng/g, 2.5 ng/g, and 10 ng/g of TTX were in the ranges of 99.5–107.2% in *T. obscurus* (Skin), 90.5–99.8% in *T. obscurus* (Muscle), and 92.9–104.0% in *T. obscurus* (Liver), respectively. Meanwhile, the recoveries of 0.5 ng/g, 2.5 ng/g and 10 ng/g were 100.1, 96.4, and 104.1% in heat-processed *Gadus*, respectively. In addition, the precisions ranged from 1.60 to 6.59% in fresh pufferfish organs and from 2.59 to 7.05% in heat-processed *Gadus*. Results indicated that the established method exhibited the good accuracy, favorable repeatability, and satisfactory feasibility, which could be applied to detect the TTX in real samples.

### 3.3. Detection of TTX in Real Samples

Table 5 and Table 6 list the results of TTX detected by the established method in different aquatic products, including the fresh and heat-processed aquatic products. Obviously, most of the collected samples were not detected TTX, which indicated the relatively safety of the fresh and heat-processed fishes with respect to TTX. Meanwhile, one of *T. obscurus* was detected 8.41 ng/g of TTX in its muscle, which was less than the safety limit value of 2 μg/g [49]. Additionally, one of heat-processed *Thamnaconus septentrionalis* was tested 219 ng/g of TTX, which was probably caused by processing mode or adulteration or confusion with other TTX-containing aquatic products. Although the TTX levels detected in the fresh and heat-processed aquatic products were not high, it is essential to pay attention to preventing acute poisoning for the relatively short incubation period of TTX.

## 4. Conclusions

In summary, a highly sensitive method was developed to simultaneously detect TTX in fresh and heat-processed aquatic products based on high-performance liquid chromatography–tandem mass spectrometry, which was investigated the chromatography column and liquid phase to get the optimal detection conditions. The results indicated that the BEH amide column exhibited an excellent performance in comparison with the common TSK amide column on the detection of TTX and the intensity of TTX decreased with the addition of ammonium acetate. The established method showed a wide detection range of 0.2–100 ng/g with a good linear dependent coefficient of 0.9998. The limit of detection was low to 0.2 ng/g. The spiked recoveries were 90.5–107.2% in the different tissues of fresh negative *Takifugu,* and 96.4–104.1% in the heat-processed *Gadus*, evidencing the excellent accuracy and the satisfactory feasibility. Furthermore, the proposed method was utilized to simultaneously detect TTX in various fresh and heat-processed aquatic products. Although the TTX levels detected in fresh and heat-processed aquatic products were not high in our study, it required to pay attention to preventing acute poisoning for the relatively short incubation period of TTX.

## Figures and Tables

**Table 1:** Gradient elution program of the target analytes.

Time/min	Mobile Phase A/%	Mobile Phase B/%	Hold Time/min
0	25	75	1.00
1.0	25	75	0.10
1.1	95	5	2.90
4.0	95	5	0.10
4.1	25	75	4.90

**Table 2:** Linear ranges, calibration curves, R^2^, LOD, LOQ, and *ME* of the developed method.

Matrix	LR (ng/g)	CV	R^2^	LOD (ng/g)	LOQ (ng/g)	*ME* (%)	Repeatability (%)
-	0.2–100	y = −5007.64 + 13988.30x	0.9998	-	-	-	-
*T. obscurus*		y = −3549.34 + 11416.6x	0.9992	0.2	0.5	18.3	5.79
Heat-processed *Gadus*		y = −2230.15 + 14082.1x	0.9992	0.2	0.5	−0.671	7.57

**Table 3:** Comparison of the TTX determination based on the proposed method with the previously reported literature using HPLC-MS/MS method.

Linear Range	LOD	Matrix	Ref.
-	3 ng/g	mangrove horseshoe crab	[19]
1.56–100 ng/mL	1.56 ng/g	shellfish	[37]
3–25,800 ng/g	6.245 ng/g	mussel	[38]
10–1000 ng/g	2.3 ng/g	pufferfish muscle	[46]
50–37,500 ng/g	410 ng/g	puffer fish	[47]
-	41 ng/g	mackerel fish extracts	[48]
0.2–100 ng/g	0.2 ng/g	pufferfish and high-processed *Gadus*	This work

**Table 4:** The determination of TTX in *T. obscurus* and heat-processed *Gadus* based on the established method.

Samples	Initial Amount (ng/g)	Spiked (ng/g)	Measured Average (ng/g)	Recovery (%)	Precision (%)
*T. obscurus* (Skin)	N.D.	0.5	0.536	107.2	3.92
	N.D.	2.5	2.657	106.3	2.45
	N.D.	10.0	9.953	99.5	6.16
*T. obscurus* (Muscle)	N.D.	0.5	0.453	90.5	1.60
	N.D.	2.5	2.373	94.9	2.40
	N.D.	10.0	9.980	99.8	6.59
*T. obscurus* (Liver)	N.D.	0.5	0.517	103.4	3.36
	N.D.	2.5	2.320	92.8	3.95
	N.D.	10.0	10.400	104.0	3.47
Heat-processed *Gadus*	N.D.	0.5	0.501	100.1	3.35
	N.D.	2.5	2.410	96.4	2.59
	N.D.	10.0	10.410	104.1	7.05

**Table 5:** Determination of TTX in different fresh pufferfishes and heat-processed fish based on the established method.

Number	Samples	Sampling City	Collection Date	Tissues	Measured * (ng/g)
1	*T. obscurus*	Zhangzhou, Fujian Province	10 January	Skin	N.D.
				Muscle	N.D.
				Liver	N.D.
2	*T. obscurus*	Zhangzhou, Fujian Province	8 April	Skin	N.D.
				Muscle	8.41 (5.71%)
3	*T. obscurus*	Taizhou, Jiangsu Province	11 May	Skin	N.D.
				Muscle	N.D.
4	*T. obscurus (1#)*	Guangzhou, Guangdong Province	12 June	Skin	N.D.
				Muscle	N.D.
				Liver	N.D.
5	*T. obscurus (2#)*	Dongguan, Guangdong Province	12 June	Skin	N.D.
				Muscle	N.D.
				Liver	N.D.
6	*T. obscurus*	Dongguan, Guangdong Province	12 June	Skin	N.D.
				Muscle	N.D.
				Liver	N.D.
7	*T. obscurus*	Yangjiang, Guangdong Province	12 June	Skin	N.D.
				Muscle	N.D.
				Liver	N.D.
8	*T. flavidus*	Shanghai	11 August	Skin	N.D.
				Muscle	N.D.
				Liver	N.D.
9	*T. rubripes (1#)*	Weihai, Shandong Province	18 September	Skin	N.D.
				Muscle	N.D.
10	*T. rubripes (2#)*	Weihai, Shandong Province	18 September	Skin	N.D.
				Muscle	N.D.
11	*T. obscurus (1#)*	Jiangyin, Jiangsu Province	11 October	Skin	N.D.
				Muscle	N.D.
12	*T. obscurus (2#)*	Jiangyin, Jiangsu Province	11 October	Skin	N.D.
				Muscle	N.D.
13	*T. obscurus (1#)*	Taizhou, Jiangsu Province	11 October	Skin	N.D.
				Muscle	N.D.
14	*T. obscurus (2#)*	Taizhou, Jiangsu Province	11 October	Skin	N.D.
				Muscle	N.D.
15	*T. obscurus*	Shanghai	26 November	Skin	N.D.
				Muscle	N.D.
16	*T. rubripes*	Tangshan, Hebei Province	3 December	Skin	N.D.
				Muscle	N.D.

* N.D.: not found (below LOD) in this work.

**Table 6:** Determination of TTX in different heat-processed fishes based on the established method.

Number	Heat-Processed Fishes	Collection Date	Measured (ng/g)
1	*Trichogaster leeri*	30 May	N.D.
2	*Gadus*	30 May	N.D.
3	*Gadus*	30 May	N.D.
4	*Trichogaster leeri*	1 June	N.D.
5	*Gadus*	1 June	N.D.
6	*Redeye mullet*	1 June	N.D.
7	*Gadus*	4 June	N.D.
8	*Anguillidae*	4 June	N.D.
9	*Gadus*	4 June	N.D.
10	*Gadus*	5 June	N.D.
11	*Gadus*	1 August	N.D.
12	*Scomberomorus niphonius*	1 August	N.D.
13	*Lophiiformes*	1 August	N.D.
14	*Gadus*	1 August	N.D.
15	*Scomberomorus niphonius*	1 August	N.D.
16	*Gadus*	1 August	N.D.
17	*Gadus*	1 August	N.D.
18	*Gadus*	1 August	N.D.
19	*Gadus*	1 August	N.D.
20	*Lophiiformes*	1 August	N.D.
21	*Rajiformes*	2 August	N.D.
22	*Anguillidae*	2 August	N.D.
23	*Gadus*	2 August	N.D.
24	*Trichogaster leeri*	2 August	N.D.
25	*Gadus*	2 August	N.D.
26	*Thamnaconus septentrionalis*	3 August	219 (1.59%)
27	*Gadus*	3 August	N.D.
28	*Gadus*	3 August	N.D.

## Data Availability

Data sharing is not applicable.

## Supplementary Materials

The following supporting information can be downloaded at: https://www.mdpi.com/article/10.3390/foods11070925/s1, Figure S1: TIC and selective product ions chromatograms of 25 ng/mL TTX.

## Sample Availability

Samples of the compounds are not available from the authors.
