# Supplementary figures and images for: Simultaneous Determination of Tetrodotoxin in the Fresh and Heat-Processed Aquatic Products by High-Performance Liquid Chromatography–Tandem Mass Spectrometry
Source: Foods. 2022 Mar 23;11(7):925. doi: 10.3390/foods11070925 (PMC8997983; doi:10.3390/foods11070925)

Supplementary Materials

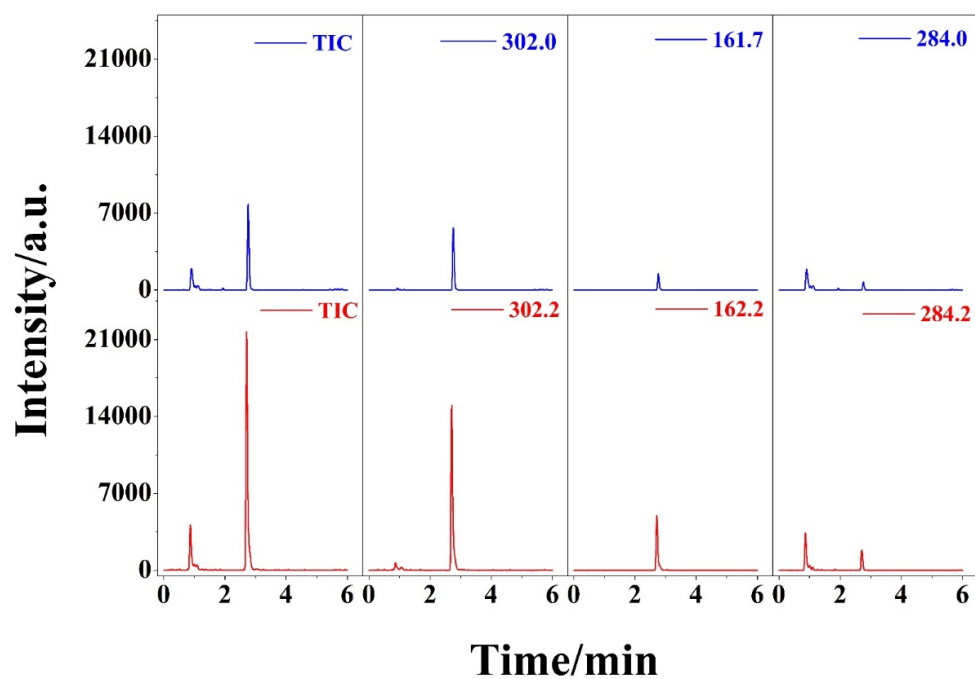

Figure S1. TIC and selective product ions chromatograms of 25 ng/mL TTX.

Supplement: Supplementary file 1 [file foods-11-00925-s001.zip › foods-1634054-supplementary.pdf]
